# Retinal Nerve Fiber Layer Thickness Progression after Robotic-Assisted Laparoscopic Radical Prostatectomy in Glaucoma Patients

**DOI:** 10.1155/2019/6576140

**Published:** 2019-12-06

**Authors:** Kazuyuki Hirooka, Kaori Ukegawa, Eri Nitta, Nobufumi Ueda, Yushi Hayashida, Hiromi Hirama, Rikiya Taoka, Yuma Sakura, Mari Yamasaki, Hiroyuki Tsunemori, Mikio Sugimoto, Yoshiaki Kiuchi

**Affiliations:** ^1^Department of Ophthalmology and Visual Science, Graduate School of Biomedical Sciences, Hiroshima University, Hiroshima 734-8551, Japan; ^2^Department of Ophthalmology, Kagawa University Faculty of Medicine, Miki, Kagawa 761-0793, Japan; ^3^Department of Urology, Kagawa University Faculty of Medicine, Miki, Kagawa 761-0793, Japan

## Abstract

**Purpose:**

To investigate the effect of the steep Trendelenburg position surgical procedure on the retinal structure and function during robotic-assisted laparoscopic radical prostatectomy (RALP) in glaucoma patients.

**Methods:**

At 1 month and 1 day before and at 1 and 2 months after the RALP operation, 10 glaucoma patients underwent standard automated perimetry and optical coherence tomography. After placing patients in a supine position, intraocular pressure (IOP) was measured at 5 min after intubation under general anesthesia (T1), at 5 discrete time points (5, 30, 60, 120, and 180 min; T2-6) and at 5 min after returning to a horizontal supine position (T7). The Guided Progression Analysis software program was used to assess serial retinal nerve fiber layer (RNFL) thicknesses and visual field progression.

**Results:**

Eight additional patients were newly diagnosed in addition to the two previous glaucoma patients. Average IOP (mmHg) at each time point was as follows: T1 = 11.2 ± 3.8, T2 = 19.0 ± 4.4, T3 = 23.3 ± 6.3, T4 = 25.1 ± 4.3, T5 = 25.5 ± 5.1, T6 = 28.3 ± 4.8, and T7 = 22.6 ± 5.4. IOP significantly increased during RALP. RNFL thickness progressed in two eyes of two patients after the surgery, even though there was no progression of the visual field.

**Conclusions:**

Two eyes of two patients exhibited significant RNFL thickness progression. Since an increased IOP during the surgery was the probable cause of the changes, ophthalmologic examinations should be performed before and after RALP, especially in glaucoma patients.

## 1. Introduction

One of the most common cancers in men is prostate cancer. Since the initial use in 2000 by Menon et al. [1], robot-assisted laparoscopic radical prostatectomy (RALP) has been increasingly used, with the procedure now being the predominant technique for the surgical management of prostate cancer in 85% of all prostatectomies [2]. In fact, due to multiple benefits, such as improved functional outcomes, reduced blood loss, fewer perioperative complications, and a faster return to work, RALP has now replaced the open prostatectomy procedure [3–5]. In order to perform this procedure, the use of a steep Trendelenburg position is required with the patients in a supine position with their feet placed above their head at a 25 to 30 degree angle of inclination. However, hemodynamic issues are sometimes known to be associated with the use of the steep Trendelenburg position, such as an increased intraocular pressure (IOP).

In order to be able to maintain both the eye structure and physiology, the IOP is an essential factor. Furthermore, previous studies have reported finding a correlation between intraoperative IOP changes and the clinical postoperative ophthalmological outcomes [6–8]. Taketani et al. examined visual field defects at 1 week after surgery using optical coherence tomography (OCT) and reported finding that the visual field returned to normal within 3 months after the surgeries in all cases, even though there were no abnormal findings in the fundus, retinal nerve fiber layer (RNFL) thickness, or optic disc morphology [7]. In contrast, we recently reported that despite all patients having an intraoperative increase in the IOP, their pre- and postoperative observations showed that none of these patients had any significant changes in the retinal structure and function [8].

The major risk factor for glaucoma progression is elevated IOP [9]. However, as compared with the preoperative IOP value, 1 hour after the use of a steep Trendelenburg position, a statistically significant increase of 5–16 mmHg has been reported to occur [10]. Therefore, complications due to increases in the IOP during the surgical procedure could lead to serious visual loss in glaucoma patients.

The present study examined glaucoma patients and attempted to determine the effect of RALP on the retinal function and structure. Static automated perimetry and optical OCT were used to evaluate the visual field and RNFL thickness before and after RALP.

## 2. Materials and Methods

### 2.1. Patients

This single-center, prospective, nonrandomized study was conducted in accordance with the principles outlined in the Declaration of Helsinki. The study protocol was approved by the Ethics Committee of Kagawa University Faculty of Medicine. Written informed consent was provided by each subject after explanation of the study protocol. All patients, approximately 200 patients, scheduled for RALP visited our ophthalmology department. A complete ophthalmic examination was performed, and 10 patients were diagnosed open-angle glaucoma. A total of 10 consecutive male open-angle glaucoma patients who underwent the RALP procedure were enrolled in the study between July 2016 and August 2018 at Kagawa University Hospital. At 1 month and 1 day prior and at 1 and 2 months after the operation, all enrolled patients underwent ophthalmic evaluations at our Ophthalmology Department. Slit-lamp and indirect ophthalmoscopy were used for the dilated fundus examination with stereoscopic biomicroscopy of the optic nerve head, while a Goldman applanation tonometer was used to measure the IOP, and visual acuity testing was performed with refraction. Subjects also underwent gonioscopic examinations that were analyzed and evaluated according to the Shaffer classification. Patients found to have a history of any kind of neurologic disease, with the exception of glaucoma, retinal laser procedure, retinal pathology, or retinal surgery, were excluded from the study. Visual field and OCT testing were performed in all enrolled subjects.

### 2.2. Cirrus HD-OCT RNFL Thickness and Optic Disc Morphology

Cirrus HD-OCT (Carl Zeiss Meditec, Dublin, CA) was used to obtain all measurements. This device is based on the use of spectral domain technology, which uses an optic disc cube that is generated from a 3-dimensional dataset. Each of the datasets was composed of 200 A-scans. These scans were derived from 200 B-scans that were obtained over a 6 mm^2^ area that was centered on the optic disc. This cube was then used to create an RNFL thickness map. The center of the disk was determined by the software. Subsequently, the software used the dataset to extract a circumpapillary circle (1.73 mm radius). Signal strengths of at least 6 were required in order for the data to be included in the analysis of the current study. The OCT instrument automatically determined the RNFL thickness deviation and the RNFL thickness change maps. The data were then exported to a computer for the purpose of analyzing the progression pattern of the RNFL defects. To visualize the RNFL defects, the RNFL thickness deviation map, which was composed of 50 × 50 pixels, was used. Yellow was used to code the pixel when the RNFL measurements were below the lower 95% of the percentile range for a particular pixel, while red was used if it was below 99%.

Subjects underwent RNFL measurements at 1 month and 1 day prior and at 1 and 2 months after the operation. RNFL thickness changes were mapped using one of the components of the GPA software (Carl Zeiss Meditec). Using the serial RNFL thickness maps, this software is able to create an event-based analysis of the RNFL progression. In order to ensure that changes at the same pixel locations can be measured, the software automatically aligns and registers the baseline and follow-up OCT images. However, a minimum of four patient visits is required in order to generate a GPA report. After obtaining and analyzing the images, the images are overlaid by the GPA program with the serial RNFL thickness compared versus that obtained during the duration of the follow-up. The RNFL thickness progression was assessed using the event analysis of the GPA software. Baseline values were calculated based on the data obtained and averaged from the first two exams. After completing the series of RNFL thickness measurements, the GPA software was used to compare the baseline RNFL thickness values to the final measurement values. When the RNFL Thickness Map Progression indicated a “likely loss” or “possible loss,” this was defined as progression of the RNFL thickness.

### 2.3. Visual Field Examination

At 1 month and 1 day prior to and at 1 and 2 months after the operation, all patients underwent standard visual field testing. A static automated white-on-white threshold perimetry (Humphrey Field Analyzer II; Carl Zeiss Meditec) with the 30-2 SITA (Swedish Interactive Threshold Algorithm) standard test was used to perform the visual field testing. If the fixation losses and the false-positive and false-negative rates were less than 20%, the visual fields were defined as being reliable. Our current analysis only used reliable test data.

The GPA software was used to analyze and compare the visual field data collected during the series of follow-up examinations with the baseline visual field data for the patients. After averaging the data from the first two exams, these values were used as the baseline and then compared with the progression evaluation. After evaluating a stable group of glaucoma patients who were tested over a very short period of time, the collected data were compared with the observed modifications of the threshold in order to evaluate the progression. These evaluations took into account the fluctuations related to the eccentricity and advancing disease. Consecutive visual field tests were performed and examined at the same locations (≥3) in order to determine if progression had occurred. As per the findings of a prior study [11], if progression was determined to have occurred in 2 locations, the GPA printouts defined these results as “possible progression.” When 3 locations were found, this was defined as “likely progression.”

### 2.4. General Anesthesia Procedures

Using a standardized anesthesia protocol, all patients were anesthetized during the surgical procedures with 2–4 *μ*g/mL propofol or 3–5% desflurane. In order to maintain the blood pressure, heart rate, and bispectral index, all patients additionally received a continuous infusion of 0.1–1.0 *μ*g/kg/min remifentanil. Sevoflurane concentration and dosing of fentanyl were adjusted to maintain the mean arterial blood pressure within 20% of the preoperative pressure. Remifentanil and fentanyl were administered for pain relief, while muscle relaxation was achieved through the use of rocuronium. To maintain an end-tidal carbon dioxide (ETCO_2_) concentration of 30–40 mmHg, mechanical ventilation of the lung was used.

IOP measurements in both eyes of each patient were measured using a Tono-Pen XL handheld tonometer (Medtronic, Jacksonville, FL) on the day of the operation. The tonometer was calibrated in accordance with the guidelines of the manufacturer prior to each of the measurements. Measurements were repeated when the variability between the sequential measurements exceeded 5%. After placing the patients under systemic anesthesia (T1) and in a supine position, IOP measurements were performed at 5 min after intubation, then at 6 discrete time points (5, 30, 60, 120, and 180 min; T2–6) after the head was lowered 30 degrees, and then finally at 5 min after returning the patients to a horizontal supine position (T7). IOP measurements in each subject were all performed by the same examiner.

### 2.5. Statistical Analysis

For all of the data analyses, Dunnett's multiple comparison test was used. All statistical analyses were performed using SPSS version 19.0 (IBM, New York, NY). A *P* value of less than 0.05 was considered to be statistically significant. Data are presented as mean ± standard deviation.

## 3. Results

A total of 20 eyes of 10 subjects, aged 63 to 81 years, were included in the study, with all of these patients found to have open angles (grade 3 and 4 according to the Shaffer grading system). In addition to the two previously diagnosed patients, eight other patients were newly diagnosed with glaucoma.

**Figure 1:**
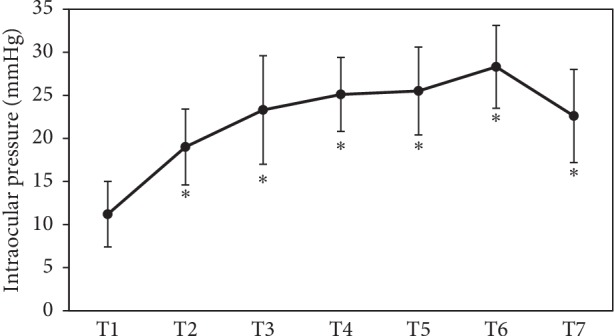
Intraocular pressure (IOP) at each time point. IOP was increased during RALP. ^*∗*^*P* < 0.001 compared with T1.

Mean operation time was 227.3 ± 47.9 min (range: 167–345 min). Mean blood loss was 90 ± 131.7 mL (range: 0–440 mL). During the surgery, blood pressure was measured every hour.  Table 1 shows each patient result of blood pressure at each measure points. Mean blood pressure was 92.9 ± 16.9/58.5 ± 15.5 mmHg at T1, 108.6 ± 13.0/64.1 ± 9.2 mmHg at T4, 101.0 ± 12.5/60.0 ± 6.3 mmHg at T5, 103.0 ± 11.9/59.1 ± 7.8 mmHg at T6, and 91.5 ± 10.6/53.5 ± 3.5 mmHg at T7. No significant differences in blood pressure level were detected between T1 and other points (T4–7) (*P* > 0.05).  Table 2 shows each patient result of IOP at each measure points. Mean IOP was 11.2 ± 3.8 mmHg (range: 7–22 mmHg; *n* = 20) at T1, 19.0 ± 4.4 mmHg (range: 12–26 mmHg; *n* = 20) at T2, 23.3 ± 6.3 mmHg (range: 14–41 mmHg; *n* = 20) at T3, 25.1 ± 4.3 mmHg (range: 18–34 mmHg; *n* = 20) at T4, 25.5 ± 5.1 mmHg (range: 16–36 mmHg; *n* = 20) at T5, 28.3 ± 4.8 mmHg (range: 24–39 mmHg; *n* = 8) at T6, and 22.6 ± 5.4 mmHg (range: 14–33 mmHg; *n* = 20) at T7 (Figure 1). A significantly increased IOP was observed at all points (T2–7) as compared with T1 (*P* < 0.001).

Mean RNFL thickness was 76.9 ± 10.5 *μ*m at 1 month before surgery, 78.1 ± 11.7 *μ*m at 1 day before surgery, 78.3 ± 10.3 *μ*m at 1 month after surgery, and 77.3 ± 11.2 *μ*m at 2 months after surgery, respectively (Table 3). After RALP, no visual field progression was observed. Mean deviation (MD) was −4.30 ± 4.03 dB at 1 month before surgery, −3.93 ± 4.41 dB at 1 day before surgery, −3.73 ± 4.67 dB at 1 month after surgery, and −3.56 ± 4.72 dB at 2 months after surgery, respectively (Table 3).

**Figure 2:**
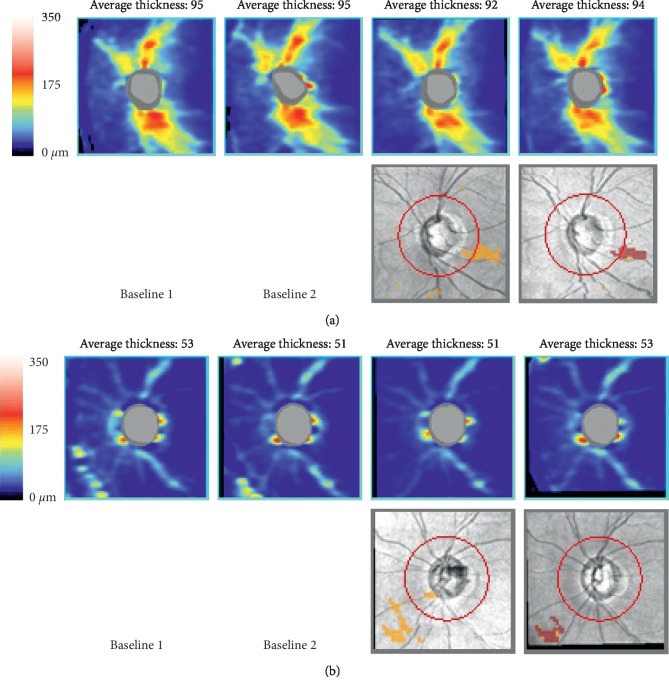
Progressive thinning of the retinal nerve fiber layer (RNFL). Guided Progression analysis (GPA) detected progression of RNFL thinning. (a) 71-year-old man with open-angle glaucoma. (b) 68-year-old man with open-angle glaucoma.

Table 4 showed individual data of RNFL thickness at each measure points. RNFL thickness progression was observed in two eyes of two patients after the RALP. Progressive RNFL thinning was observed in a 71-year-old man with open-angle glaucoma (Patient no. 2, left eye) (Figure 2(a)). IOP was 10 mmHg at T1, 13 mmHg at T2, 14 mmHg at T3, 20 mmHg at T4, 20 mmHg at T5, 19 mmHg at T6, and 19 mmHg at T7. Operation time was 206 min. Progressive RNFL thinning was also observed in a 68-year-old man with open-angle glaucoma (Patient no. 3, left eye) (Figure 2(b)). IOP was 13 mmHg at T1, 25 mmHg at T2, 41 mmHg at T3, 34 mmHg at T4, 36 mmHg at T5, 33 mmHg at T6, and 27 mmHg at T7. The total operation time was 208 min.

## 4. Discussion

To the best of our knowledge, this is the first report to evaluate glaucoma patients undergoing RALP and determine the effect on the retinal structure and function. No visual field changes were observed even though the RNFL thickness progressed in two eyes of two patients.

Taketani et al. examined 50 eyes (25 patients) and reported finding significant visual field defects at one week after RALP in 7 eyes, with the visual field returning to normal within 3 months [7]. In contrast, OCT evaluations of 7 eyes indicated that there were no abnormal findings for either the RNFL thickness or the optic disc morphology. Therefore, these authors concluded that the retinal pathologic changes were unlikely to be due to transient visual field defects. We recently examined normal subjects and reported that pre- and postoperative observations revealed no significant changes in the retinal structure and function [8]. Mathew et al. also evaluated normal subjects at 1 month postoperatively and reported that none of the patients developed visual field or RNFL changes [12]. Therefore, the use of the steep Trendelenburg position during time-limited procedures in patients without any pre-existing disease might have little or no risk of developing an increased IOP. However, due to the already damaged retinal structure and function that is seen in glaucoma patients, greater attention needs to be paid to glaucoma patients before and after RALP.

During the use of the steep Trendelenburg position, there is actual elevation of the episcleral venous pressure that impairs the facility of the aqueous outflow [13]. Molloy et al. administered dorzolamide-timolol drops immediately following induction of anesthesia and reported finding a significantly reduced evaluation in the IOP in patients undergoing RALP when using the steep Trendelenburg position [14]. However, there was no IOP reduction noted during RALP, following the administration of brimonidine 30 minutes before the start of the surgery [12]. Furthermore, it has also been reported that there was attenuation of the increase in the IOP during RALP after the systemic administration of dexmedetomidine before induction of general anesthesia [15]. In addition, there have been no studies that have examined reductions in the IOP after the use of a prostaglandin analogue in patients placed in the steep Trendelenburg position. Moreover, there is also the possibility that increases in the episcleral venous pressure might not have as great of an effect on increases in the uveoscleral outflow as compared with those caused by suppression of the aqueous humor production. It has also been reported that the application of ocular hypotensive eyedrops, such as timolol, latanoprost, or brinzolamide, does not alter the postural response of the IOP in glaucoma patients [16]. Therefore, further studies that examine whether dorzolamide-timolol or dexmedetomidine can effectively reduce the elevated IOP during the steep Trendelenburg position in glaucoma patients will need to be undertaken.

Another important finding in our current study was that we newly diagnosed glaucoma in 8 out of 10 patients during our presurgical ophthalmic examinations. In the Tajimi study, only 8 out of 119 primary open-angle glaucoma (POAG) patients (6.7%) had been previously diagnosed with POAG [17]. Therefore, if the steep Trendelenburg position surgical procedure is to be used, it is important that all of these patients first undergo screening during their ophthalmic examinations to determine if glaucoma might be present.

An anesthetic duration of more than 6 hours has been reported by the American Society of Anesthesiologists registry to be a strong risk factor for postoperative vision loss [18]. Furthermore, Molloy et al. have recommended the use of intraoperative “rest stops” at the 4-hour time point. In our current study, the mean operation time was 227.3 min [14]. Moreover, the operation time in the eyes of the two cases with progression of RNFL thickness was 206 or 208 min, respectively. While the mean IOP in the 71-year-old patient during the surgery was 16.8 mmHg, it was 34.0 mmHg during surgery in the 68-year-old patient. This could be important, as one of the risk factors for progression of RNFL thinning is an increased IOP during surgery.

There were a couple of limitations for our current study. First, although we performed ophthalmic examinations at 1 and 2 months postoperatively, there is a possibility that a reversible adverse effect could have occurred within that first 1-month time period. Second, the small sample size was also another potential limitation of our current study.

In conclusion, while it remains unknown whether eyedrops can effectively reduce elevated IOP during surgery in glaucoma patients, glaucoma patients should be instructed to take their medications prior to RALP when using the steep Trendelenburg position and have their IOP routinely monitored during these surgeries. Furthermore, in patients scheduled to undergo RALP, comprehensive eye examinations should be recommended in all of these subjects prior to the surgical procedure.

## Figures and Tables

**Table 1:** Individual data for IOP changes.

Patient	Age (yrs)	R/L	IOP (mmHg)							
			T1	T2	T3	T4	T5	T6	T7	T8
1	67	R	10	17	24	21	21			
		L	10	19	21	22	23			
2	71	R	10	20	20	18	24			
		L	10	13	14	20	20			
3	68	R	10	26	36	32	36			
		L	13	25	41	34	36			
4	76	R	15	25	22	25	27	39		
		L	13	24	21	26	28	31		
5	72	R	7	14	19	21	25	27		
		L	8	15	21	23	24	28		
6	63	R	9	14	23	26	24			
		L	8	12	24	33	22			
7	72	R	13	21	25	23	25			
		L	18	22	29	27	29			
8	74	R	13	20	26	26	23	26	27	25
		L	22	20	22	27	23	24	25	17
9	81	R	10	17	16	25	34			
		L	9	13	19	23	23			
10	69	R	7	20	19	22	27	26		
		L	8	23	23	28	16	25		

IOP: intraocular pressure, R: right eye, L: left eye.

**Table 2:** Individual data for blood pressure changes.

Patient	Systolic/Diastolic blood pressure (mmHg)						
	T1	T3	T4	T5	T6	T7	T8
1	76/56		89/66	88/59	85/59		
2	80/53		104/65	96/66	92/60		
3	120/99	145/94	122/80	107/68	112/73		
4	71/44		135/78		121/66		
5	107/61	120/72	100/56	109/60	103/51		
6	115/63		98/61	85/48			
7	81/47		108/64	115/63	118/56		
8	100/60	111/66	108/64	92/52	97/61	84/51	107/66
9	92/52		107/56	96/61	98/60		
10	87/50		115/51	121/59	101/46	99/56	

**Table 3:** Retinal nerve fiber layer thickness and visual field at each measure point.

	Average RNFLT (*μ*m)	*P* value	Average MD (dB)	*P* value
1 M before	76.9 ± 10.5		−4.30 ± 4.03	
1 D before	78.1 ± 11.7		−3.93 ± 4.41	
1 M after	78.3 ± 10.3	0.996	−3.73 ± 4.67	0.987
2 M after	77.3 ± 11.1	0.967	−3.56 ± 4.72	0.956

M: month, D: day, RNFLT: retinal nerve fiber layer thickness, MD: mean deviation. *P* value is compared with 1 D before.

**Table 4:** Individual data of retinal nerve fiber layer thickness at each measure point.

Patient	R/L	RNFLT (*μ*m)			
		1 M before	1 D before	1 M after	2 M after
1	R	82	82	79	80
	L	90	98	93	93
2	R	94	94	94	94
	L	94	95	92	94
3	R	88	91	92	90
	L	53	51	51	53
4	R	79	79	80	82
	L	71	75	70	69
5	R	69	71	72	71
	L	74	73	75	71
6	R	62	63	72	64
	L	71	67	82	73
7	R	74	71	72	64
	L	68	77	74	73
8	R	74	74	74	77
	L	86	89	87	87
9	R	74	78	76	78
	L	73	69	72	69
10	R	78	80	75	80
	L	83	84	84	84

R: right eye, L: left eye, RNFLT: retinal nerve fiber layer thickness. M: month, D: day.

## Data Availability

The data (Tables and Figure) used to support the findings of this study are included within the article.
